# *Plasmodium falciparum Plasmodium* helical interspersed subtelomeric proteins contribute to cytoadherence and anchor *P. falciparum* erythrocyte membrane protein 1 to the host cell cytoskeleton

**DOI:** 10.1111/cmi.12583

**Published:** 2016-04-26

**Authors:** Alexander Oberli, Laura Zurbrügg, Sebastian Rusch, Françoise Brand, Madeleine E. Butler, Jemma L. Day, Erin E. Cutts, Thomas Lavstsen, Ioannis Vakonakis, Hans‐Peter Beck

**Affiliations:** ^1^ Swiss Tropical and Public Health InstituteBaselSwitzerland; ^2^ University of BaselBaselSwitzerland; ^3^ Department of BiochemistryUniversity of OxfordOxfordUK; ^4^ Centre for Medical Parasitology, Department of International Health, Immunology, and MicrobiologyUniversity of CopenhagenCopenhagenDenmark; ^5^ Department of Infectious DiseasesRigshospitaletCopenhagenDenmark

## Abstract

Adherence of *Plasmodium falciparum*‐infected erythrocytes to host endothelium is conferred through the parasite‐derived virulence factor *P. falciparum* erythrocyte membrane protein 1 (PfEMP1), the major contributor to malaria severity. PfEMP1 located at knob structures on the erythrocyte surface is anchored to the cytoskeleton, and the *Plasmodium* helical interspersed subtelomeric (PHIST) gene family plays a role in many host cell modifications including binding the intracellular domain of PfEMP1. Here, we show that conditional reduction of the PHIST protein PFE1605w strongly reduces adhesion of infected erythrocytes to the endothelial receptor CD36. Adhesion to other endothelial receptors was less affected or even unaltered by PFE1605w depletion, suggesting that PHIST proteins might be optimized for subsets of PfEMP1 variants. PFE1605w does not play a role in PfEMP1 transport, but it directly interacts with both the intracellular segment of PfEMP1 and with cytoskeletal components. This is the first report of a PHIST protein interacting with key molecules of the cytoadherence complex and the host cytoskeleton, and this functional role seems to play an essential role in the pathology of *P. falciparum*.

## Introduction

After invading the human erythrocyte, the malaria parasite *Plasmodium falciparum* refurbishes its host cell dramatically. The most important changes lead to sequestration of infected cells to the microvasculature of human organs – the sole cause of morbidity and mortality in malaria tropica. These changes also allow the malaria parasite to grow in a parasitophorous vacuole inside the erythrocyte and enable nutrient uptake. The parasite invests approximately 10% of its proteome to refurbish the host cell in this way. Hundreds of exported parasite proteins fall into one of two groups (Spillman *et al*., 2015). The first group is well defined and consists of proteins containing a pentameric motif, termed PEXEL/HT (*Plasmodium* export element/host targeting signal) (Hiller *et al*., 2004; Marti *et al*., 2004), which allows the establishment of a *P. falciparum* exportome with approximately 400 proteins (Sargeant *et al*., 2006). A second group of exported proteins, which do not contain a PEXEL/HT motif or any other identifiable export motif, has also been observed (PEXEL‐negative exported proteins). It is difficult to predict the true number of PEXEL‐negative exported proteins and hence the total number of exported proteins (Heiber *et al*., 2013). The export of both groups of proteins results in profound structural and morphological changes in the erythrocyte. For example it causes the formation of electron‐dense protrusions on the erythrocyte surface, called knobs (Watermeyer *et al*., 2016), alters red blood cell (RBC) rigidity (Maier *et al*., 2008) and increases membrane permeability (Nguitragool *et al*., 2011).

A key molecule and ligand for binding infected red blood cells (iRBCs) to host cell receptors on the vascular endothelium is the *P. falciparum* erythrocyte membrane protein 1 (PfEMP1). This major parasite virulence factor is embedded in the knobs through a transmembrane helix and comprises a highly variable ectodomain and a semiconserved intracellular segment, the acidic terminal segment (ATS) (Lavstsen *et al*., 2003; Mayer *et al*., 2012). The extracellular part of PfEMP1 consists of multiple adhesion domains, enabling the infected cell to bind to host adhesins including CD36, intercellular adhesion molecule‐1 (ICAM‐1) and chondroitin sulfate A (CSA). This binding leads to iRBC sequestration within the microvasculature (Kraemer and Smith, 2006). In contrast, the cytoplasmic domain is relatively conserved and was previously thought to interact with the knob‐associated histidine‐rich protein (KAHRP) (Crabb *et al*., 1997) and, potentially, with the erythrocyte cytoskeleton components actin and spectrin (Kilejian *et al*., 1991; Waller *et al*., 1999, 2002; Oh *et al*., 2000). Recent data, however, do not support a direct ATS–KAHRP interaction but rather an ATS interaction with PHIST proteins PFI1780w and PFE1605w (Mayer *et al*., 2012; Oberli *et al*., 2014).

The proteins encoded by the *phist* multigene family are defined by the presence of a 150‐amino acid domain consisting of four consecutive α‐helices. Almost all members include a signal sequence and a PEXEL motif (Sargeant *et al*., 2006). The *phist* family underwent dramatic lineage‐specific proliferation in *P. falciparum* and is suspected of playing a major role in host cell modifications in cytoplasmic protein associations (Sargeant *et al*., 2006; Oakley *et al*., 2007; Frech and Chen, 2013). To date, only a few PHIST proteins have been partially characterized and almost no molecular functions have been assigned, despite their wide distribution within the iRBC. So far, members of the PHIST protein family have been implicated in knob formation (Maier *et al*., 2008), in altered host cell rigidity (Mills *et al*., 2007; Maier *et al*., 2008), in trafficking of and interaction with PfEMP1 (Maier *et al*., 2008; Mayer *et al*., 2012; Oberli *et al*., 2014) and in iRBC adhesion to the brain microvasculature (Daily *et al*., 2005; Claessens *et al*., 2012). Moreover, PHIST proteins have been shown to localize to the iRBC periphery (Tarr *et al*., 2014), possibly binding erythrocyte cytoskeletal components (Kilili and LaCount, 2011; Parish *et al*., 2013; Proellocks *et al*., 2014). They have also been found in detergent‐resistant membrane fractions (Sanders, 2005) and in exosomes mediating cell–cell communication (Regev‐Rudzki *et al*., 2013).

Previously, we showed that PFE1605w, another member of the PHIST protein family, is exported to knobs and binds directly to the PfEMP1 ATS domain, displaying similar temporal and spatial export as PfEMP1 (Oberli *et al*., 2014). This finding differs somewhat from those of Proellocks *et al*. (2014), who suggested an alternative localization. Fluorescence polarization experiments using PFE1605w and a set of ATS domains from different PfEMP1 molecules showed substantial variation in binding affinity, suggesting that different PHIST proteins might have been optimized for different PfEMP1 members (Oberli *et al*., 2014). Here we present the first functional analysis of a PHIST protein by using inducible downregulation of PFE1605w and also a unique controlled system that blocks PFE1605w at Maurer's clefts. Both approaches showed that reduced levels of PFE1605w within the knobs lead to strongly reduced adhesion of iRBC to endothelial receptors, but that PFE1605w plays no role in transporting PfEMP1 or its surface exposure. PFE1605w directly binds the C‐terminus of different ATS domains *in vitro* and in iRBC and interacts with components of band 3 and junctional complexes at the erythrocyte membrane. This is the first report of a functional role for PFE1605w, which anchors a variety of PfEMP1 variants to the cytoskeleton of the iRBC.

## Results

### Inducible regulation of PFE1605w

**Figure 1:**
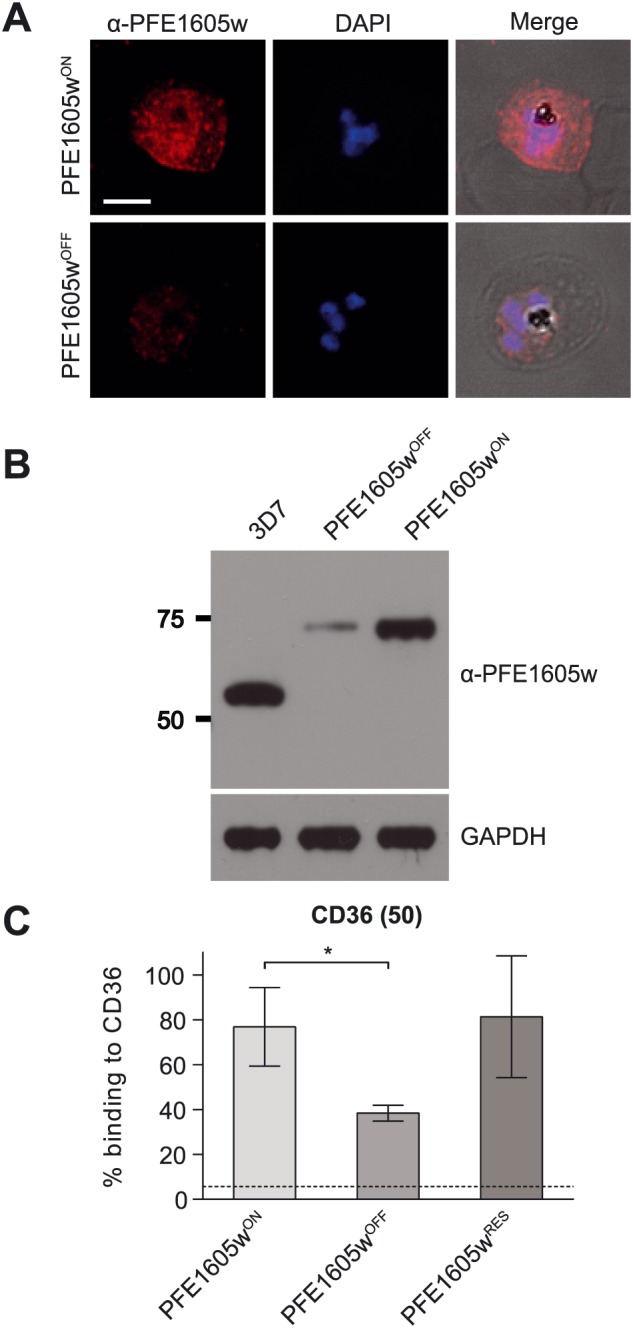
Conditional depletion of PFE1605w. Confocal immunofluorescence and Western blot analysis of synchronized 3D7 parasites expressing endogenous PFE1605w as a C‐terminally tagged DD fusion protein grown for 96 h in the presence (PFE1605w^ON^) or absence (PFE1605w^OFF^) of 625 nM Shield‐1. A. Confocal immunofluorescence. B. Western blot. The specificity of affinity‐purified polyclonal α‐PFE1605w antibodies is described in Fig. S1B. The nuclei were stained with DAPI. Scale bar = 3 µm. GAPDH was used as loading control. C. Semistatic adhesion assay of RBCs infected with PFE1605w^ON^/PFE1605w^OFF^ parasites to immobilized recombinant CD36 at 50 µg ml^−1^. The graph displays mean values across triplicate samples normalized to 3D7 wild‐type parasite binding. The error bars represent SDs of three independent experiments. An arbitrary threshold (dashed line) for unspecific binding was calculated as the mean level of iRBC binding to 1% w/v BSA plus 2 SDs. *P* values were calculated by using a two‐tailed Student's *t*‐test, asterisk indicates *P* ≤ 0.05.

To investigate the function of PFE1605w, we generated a parasite cell line that allowed a conditional expression of the endogenous protein by using the human FK506 binding protein (FKBP) destabilization domain (DD) technique (Banaszynski *et al*., 2006; Armstrong and Goldberg, 2007). For this, parasites were generated that expressed endogenous PFE1605w as a C‐terminally tagged green fluorescent protein–DD fusion protein and whose PFE1605w–DD was rapidly degraded if not stabilized by Shield‐1. The integration of the plasmid containing the coding sequence for the PFE1605w–DD construct at the correct locus was confirmed by Southern blot (Fig. S1A). Immunofluorescence assays (IFAs) showed the expected localization of tagged PFE1605w at Maurer's clefts and at the iRBC membrane (Fig. 1A) as previously shown with non‐modified protein (Oberli *et al*., 2014). In parasites grown for 96 h without Shield‐1 (PFE1605w^OFF^), PFE1605w levels were highly reduced and the residual protein was visible only within the parasite. Parasites grown for 96 h under the presence of Shield‐1 (PFE1605w^ON^) displayed normal levels and distribution of PFE1605w. Western blot analysis of synchronized PFE1605w^ON^/PFE1605w^OFF^ parasites with polyclonal antibodies against PFE1605w showed significantly reduced levels of PFE1605w in PFE1605w^OFF^ parasites compared with PFE1605w^ON^ and 3D7 wild‐type parasites (Fig. 1B). To test the PFE1605w^ON^/PFE1605w^OFF^ parasites’ adherence to recombinant CD36, a semistatic adhesion assay was performed. PFE1605w^OFF^ parasites showed a 50% (±9%, *n* = 3) decrease in adhesion to CD36 (Fig. 1C) compared with PFE1605w^ON^ parasites, a result similar to that observed with a PFE1605w gene disruption (Proellocks *et al*., 2014). Subsequent addition of Shield‐1 to the cultures (PFE1605w^RES^) restored adhesion to CD36 to the same level as that for PFE1605w^ON^ parasites (Fig. 1C). This indicates that reduced levels of exported PFE1605w results in a significant reduction of adhesion of iRBCs.

### Inducible tethering of PFE1605w at Maurer's clefts

**Figure 2:**
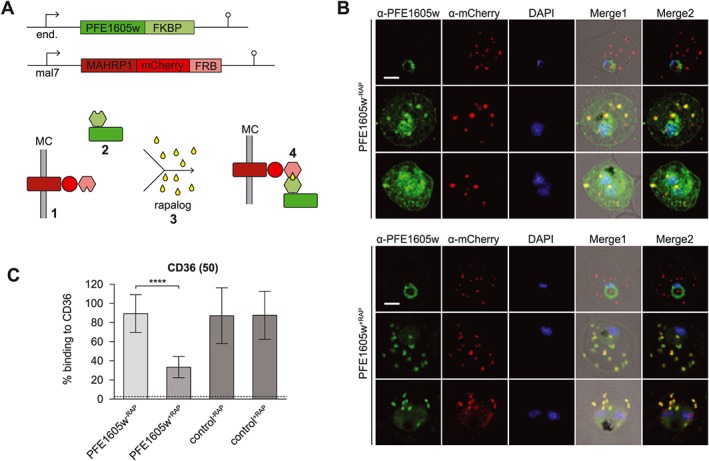
Controlled tethering of PFE1605w at Maurer's clefts. A. Schematic representation of controlled PFE1605w tethering. The episomally expressed MAHRP1‐FRB fusion protein is exported to Maurer's clefts (1) prior to the export of C‐terminally FKBP‐tagged PFE1605w (2). Upon close proximity of the two fusion proteins and addition of rapalog (3), heterodimerization of the FKBP domain and the FRB domain occurs (4) and PFE1605w is immobilized at Maurer's clefts. B. Confocal immunofluorescence analysis of parasites grown in the absence (PFE1605w^−RAP^) or presence (PFE1605w^+RAP^) of 100 nM rapalog. The nuclei were stained with DAPI. Scale bar = 2 µm. C. Semistatic adhesion assay of PFE1605w^−RAP^/PFE1605w^+RAP^ parasites to immobilized recombinant CD36 protein at 50 µg ml^−1^ concentration. The graph displays mean values across triplicate samples, and the error bars represent the SDs of three independent experiments. An arbitrary threshold (dashed line) for unspecific binding was calculated as the mean level of iRBC binding to 1% w/v BSA plus two SDs. *P* values were calculated by using a two‐tailed Student's *t*‐test; the asterisks indicate *P* ≤ 0.0001.

To confirm the importance of PFE1605w presence in knobs for cytoadherence, an alternative approach was used. By conditionally tethering PFE1605w to the cytoplasmic domain of a Maurer's cleft protein, we prevented its transport to the knobs, thereby blocking the presence of PFE1605w at the knob structure. The technique is based on the heterodimerization of the FKBP12 to the FKBP‐rapamycin binding (FRB) domain of human mechanistic target of rapamycin in the presence of rapamycin (Haruki *et al*., 2008; Busch *et al*., 2009; Robinson *et al*., 2010; Xu *et al*., 2010). First, we generated parasites that expressed PFE1605w C‐terminally fused to FKBP under the control of the endogenous promoter (Fig. S1A). These parasites were subsequently transfected with a plasmid that episomally expressed membrane‐associated histidine‐rich protein 1 (MAHRP1) fused to an mCherry tag and an FRB domain under the mal7 promoter (Figs 2A and S1C). In ring‐stage parasites, the MAHRP1–FRB fusion protein was exported to the Maurer's clefts, whereas the FKBP‐tagged PFE1605w still resided within the parasite (Fig. 2B). In trophozoite and schizont parasites, PFE1605w–FKBP was correctly exported to Maurer's clefts and to knobs as previously described (Oberli *et al*., 2014). Upon adding 100 nM rapalog (a rapamycin analogue) to ring‐stage parasites, a ternary complex at the Maurer's clefts composed of MAHRP1–FRB, rapalog and PFE1605w–FKBP was formed as soon as PFE1605w–FKBP was exported to Maurer's clefts (Fig. 2B) and PFE1605w–FKBP was blocked from localizing in the knobs. Next, we tested the cytoadhesive properties of parasites grown in the presence or absence of rapalog (PFE1605w^+RAP^/PFE1605w^−RAP^) to recombinant CD36. Parasites cultured in the presence of rapalog (PFE1605w^+RAP^) showed a 62% (±9%, *n* = 3) reduction in binding to CD36 compared with parasites grown without rapalog (PFE1605w^‐RAP^) (Fig. 2C). To demonstrate that endogenous untagged PFE1605w does not bind MAHRP1–FRP upon addition of the rapalogue, 3D7 wild‐type parasites were transfected with the MAHRP1–FRB plasmid. In both cases (control^+RAP^/control^−RAP^), the parasites showed comparable levels of binding to CD36, indicating that no heterodimerization occurred (Fig. 2C).

### *PFE1605w has no significant role in* P. falciparum *erythrocyte membrane protein 1 transport*

To test whether PFE1605w reduction or tethering impairs transport of other well‐characterized exported proteins, we analysed the PFE1605w^ON^/PFE1605w^OFF^/PFE1605w^+RAP^/PFE1605w^−RAP^ parasites by IFA by using antibodies against PfEMP1, PfEMP3, KAHRP, mature parasite‐infected erythrocyte surface antigen (MESA), ring‐infected erythrocyte surface antigen (RESA), MAHRP1, MAHRP2 and HSP70x. All tested proteins revealed correct subcellular localization in parasites (data shown for PFE1605w^ON^/PFE1605w^OFF^ parasites; Fig. S2).

**Figure 3:**
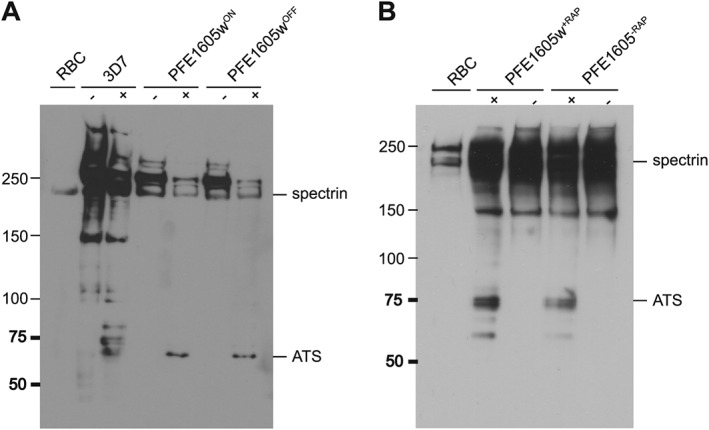
PfEMP1 surface exposure is not impaired upon PFE1605w depletion in knobs. Determination of PfEMP1 surface exposure by detecting trypsin cleavage in 3D7 wild type, PFE1605w^ON^/PFE1605w^OFF^ and PFE1605w^+RAP^/PFE1605w^−RAP^ parasites. A. PFE1605w^ON^/PFE1605w^OFF^. B. PFE1605w^+RAP^/PFE1605w^−RAP^ parasites. Trophozoites of each parasite line were treated with trypsin (+) or without trypsin (−), and the extracts were analysed by Western blot by using antibodies against ATS domains.

To test whether the observed reduction in CD36 binding was due to a reduction of PfEMP1 surface exposure, we treated iRBCs with trypsin. In all parasite cultures, PfEMP1 was correctly displayed on the iRBC surface (Fig. 3A and B) as evident by PfEMP1 proteolysis that yields intact ATS domains. The trypsin cleavage assay also revealed that the size of PfEMP1 in all parasites were identical, suggesting that the same PfEMP1 variant was expressed in the parasite lines compared. Scanning electron microscopy (SEM) showed the presence of knobs in all parasite cell lines (Fig. S3); thus, the reduction in cytoadherence observed in PFE1605w^OFF^ and PFE1605w^+RAP^ parasites was not due to decreased knob formation.

### Cytoadhesive properties of infected red blood cells expressing different *P. falciparum* erythrocyte membrane protein 1 in the absence of PFE1605w

**Figure 4:**
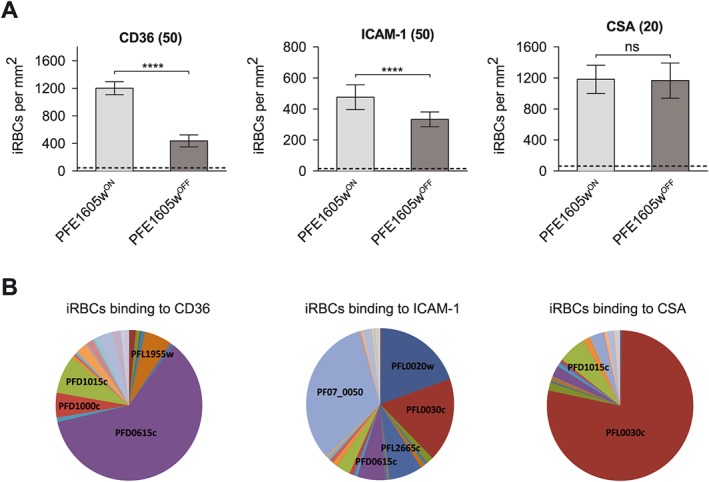
iRBCs expressing a different PfEMP1 variant show different level of reduction in cytoadherence upon conditional depletion of PFE1605w. A. Preselected iRBCs binding to either recombinant CD36, ICAM‐1 or CSA immobilized on tissue‐treated glass slides at 50 µg ml^−1^ (CD36, ICAM‐1) or 20 µg ml^−1^ (CSA) concentrations. Parasites expressing PFE1605w as a C‐terminally tagged DD fusion protein were grown for 96 h in the presence (PFE1605w^ON^) or absence (PFE1605w^OFF^) of 625 nM Shield‐1. The graphs display overall mean values across triplicate experiments using linear regression with a random effect for experiment. The error bars represent the SDs of the triplicate experiments. An arbitrary threshold (dashed line) for unspecific binding was calculated as the mean level of iRBC binding to 1% w/v BSA plus two SDs. *P* values were calculated by using a two‐tailed Student's *t*‐test. The asterisks indicate *P* ≤ 0.0001. ‘ns’ indicates *P* ≥ 0.05. B. Pie charts show the *var* transcript distribution in the selected lines. qPCR was performed with specific primers for each *var* gene as previously reported.

Previously, we showed that the recombinant PHIST domain of PFE1605w interacted with six different ATS variants with up to 25‐fold differences in affinity (Oberli *et al*., 2014). This suggested that PFE1605w might be optimized for binding to a subset of PfEMP1 variants; hence, it might be relevant only for iRBC binding to a subset of endothelial receptors. Therefore, we selected parasites expressing PFE1605w–DD on different host receptors, including CD36, ICAM‐1 and CSA, in order to isolate parasites expressing different PfEMP1 molecules. After four rounds of pre‐selection, we obtained parasites binding to CD36, ICAM‐1, or CSA (Fig. 4). Expression of *var* genes in all the pre‐selected parasite lines was tested by quantitative PCR (qPCR) and showed a clear differential expression of *var* genes, suggesting the display of a distinct PfEMP1 variant on the iRBC surface (Fig. 4B). Pre‐selected parasites were grown with and without Shield‐1 and after 96 h they were allowed to bind to their respective receptor in a semistatic adhesion assay. Parasites grown in the absence of Shield‐1 showed an approximately 64% reduction in binding to CD36 (Fig. 4A). Binding to ICAM‐1 was reduced by 30% and binding to CSA showed no reduction at all (Fig. 4A), indicating that PFE1605w plays no role in CSA‐mediated cytoadherence.

### PFE1605w binds the C‐terminal part of the acidic terminal segment

**Figure 5:**
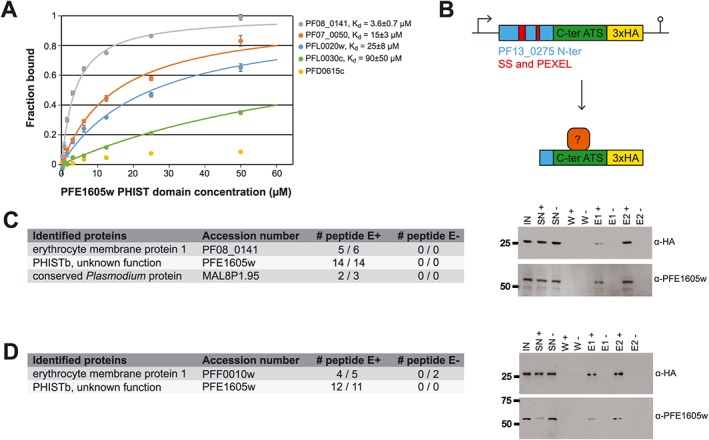
PFE1605w directly binds the ATS C‐terminus. A. Fluorescence polarization titrations of 5‐FAM‐labelled ATS‐C constructs from PfEMP1 variants (Fig. S4A) with unlabelled PFE1605w PHIST domain. Data points, normalized to the fraction of ATS‐C bound at each PFE1605w concentration, are shown as coloured circles. The error bars were derived from four technical replicates. The solid lines correspond to data fitted with a single‐site association model. Equilibrium dissociation constants (*K*_d_) for the PFE1605w–ATS‐C interaction are shown. The interaction of PfEMP1 variant PFD0615c with PFE1605w could not be fitted. B. Schematic representation of the mini‐PfEMP1 construct for Co‐IP experiments. C and D. LC‐ESI‐MS/MS results of two independent Co‐IP experiments using parasites expressing mini‐PfEMP1 constructs composed of the C‐terminal part of PF08_0141 (C) or PFF0010w (D). Only peptide hits detected in both of the duplicate experiments are shown. Samples were also analysed by Western blot with α‐HA and α‐PFE1605w antibodies. IN, input; SN, supernatant; W, wash; E, elution.

Previously, we showed that the recombinant PHIST domain of PFE1605w binds with low‐micromolar affinity to the C‐terminal part of the ATS domain (ATS‐C) of PfEMP1 variant PF08_0141 (Oberli *et al*., 2014). Sequence conservation among ATS domains suggested that ATS‐C provides the PFE1605w binding epitope in most PfEMP1 variants. To test this, we performed *in vitro* fluorescence polarization binding experiments by using the PFE1605w PHIST domain and fluorescein‐labelled recombinant ATS‐C fragments from PfEMP1 variants dominantly expressed in preselected parasites (Figs 4B and S4A). In almost all cases, we observed direct PFE1605w–ATS‐C binding with dissociation constants (*K*_d_) in the 4–90 μM range (Fig. 5A).

To test whether PFE1605w binds to ATS‐C in *P. falciparum* iRBCs, we designed two mini‐PfEMP1 constructs consisting of an N‐terminal part of a PEXEL protein (PF13_0275) including a signal sequence and a PEXEL motif, the ATS‐C of two PfEMP1 variants and a 3xHA tag to allow detection (Figs 5B and S4B). The PfEMP1 variants selected, PF08_0141 and PFF0010w, display approximately 13‐fold difference in *in vitro* affinity (5 and 65 μM *K*_d_ respectively) for the PHIST domain of PFE1605w (Oberli *et al*., 2014). Because the fusion proteins were expressed under the *crt* promoter, they were found early in the life cycle. Due to the lack of a TM domain, the mini‐PfEMP1 was soluble and exported to the erythrocyte cytosol with the predicted size (Fig. S1D). Potential ATS‐C interaction partners were detected by co‐immunoprecipitation (Co‐IP) followed by mass spectrometry (MS) for protein identification. Trophozoite extracts from parasites expressing a mini‐PfEMP1 fusion protein were used to isolate potential interacting proteins through an hemagglutinin (HA)‐affinity matrix. As a negative control, parasite extract with an excess of soluble HA peptide was added during the affinity‐matrix binding of the mini‐PfEMP1 fusion proteins. Western blot analysis confirmed that the mini‐PfEMP1 fusion proteins were successfully purified and that PFE1605w was coeluted with both mini‐PfEMP1 constructs (Fig. 5C and D). In addition, from duplicate Co‐IP experiments, the liquid chromatography–mass spectrometry (LC‐MS)/MS analysis detected from both mini‐PfEMP1 constructs more than 10 peptide hits for PFE1605w (Fig. 5C and D). These results demonstrate a direct protein–protein interaction of PFE1605w with the C‐terminal part of the ATS domain of different PfEMP1 variants.

### Potential PFE1605w interaction partners

**Figure 6:**
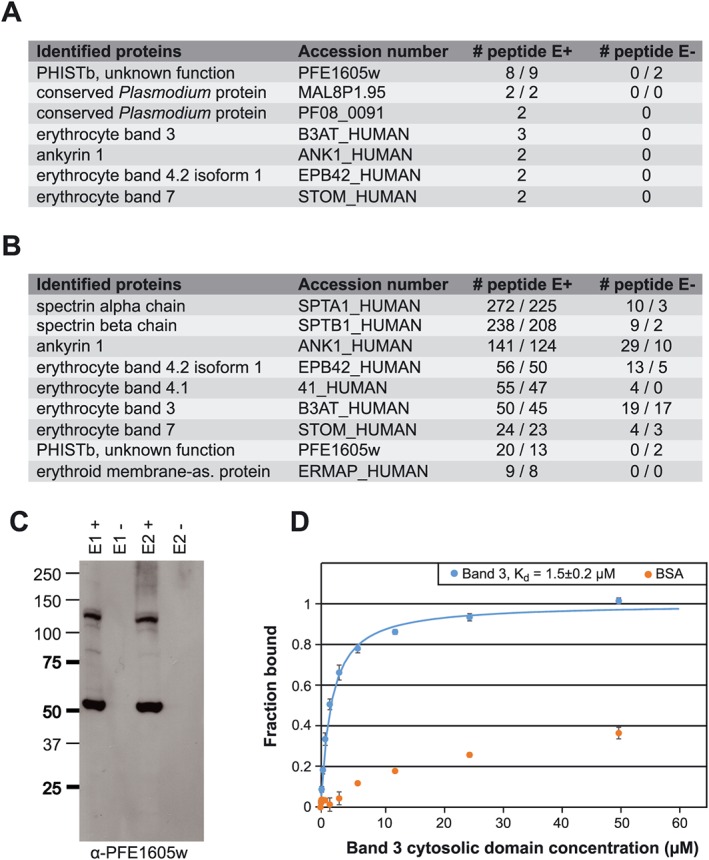
PFE1605w binds to the RBC cytoskeleton. A. LC‐ESI‐MS/MS results of two independent Co‐IP experiments using parasites expressing PFE1605w‐HA. B. LC‐ESI‐MS/MS results of two independent reverse Co‐IP experiments with α‐band 4.2 antibodies coupled to protein G Dynabeads. All experiments were performed twice. C. Elution fractions of the reverse Co‐IP experiment were also analysed by Western blot with α‐PFE1605w antibodies. D. Fluorescence polarization titrations of 5‐FAM‐labelled PFE1605w‐C with unlabelled band 3 cytosolic domain or BSA as a negative control. Data points, normalized to the fraction of PFE1605w‐C bound at each titrant concentration, are shown as coloured circles. The error bars were derived from three replicates. The fit to a single‐site association model is shown as solid line. The interaction of PFE1605w‐C with BSA could not be fitted.

To detect other potential PFE1605w interaction partners, we performed Co‐IP experiments with parasites expressing the PFE1605w–3xHA fusion protein, followed by MS‐based protein identification. Different components of the human erythrocyte cytoskeleton were detected, in addition to two *Plasmodium* proteins of unknown function (Fig. 6A).

To confirm these potential PFE1605w interaction partners from the human cytoskeleton, we performed reverse Co‐IP experiments with 3D7 wild‐type parasite lysate and specific antibodies against human bands 3 and 4.2, both of which locate equally at band 3 and junctional complexes (Mankelow *et al*., 2012), and ankyrin 1. From the elution of reverse Co‐IP with antibodies against human band 4.2, Western blots detected a dominant band, identified as PFE1605w (Fig. 6C). MS identified other components of band 3 and junctional complexes, including band 3, band 4.2, band 4.1, the α‐ and β‐chains of spectrin and ankyrin, but no other *Plasmodium* protein except PFE1605w (Fig. 6B), again suggesting that PFE1605w interacts with one or several cytoskeletal components.

To further probe the cytoskeletal interactions of PFE1605w, we produced a fluorescein‐labelled recombinant PFE1605w fragment (PFE1605w‐C) comprising the C‐terminal tail of this protein that follows the PHIST domain. PFE1605w‐C was previously shown to bind to inside‐out vesicles prepared from uninfected erythrocytes (Proellocks *et al*., 2014). In the fluorescence–polarization binding experiments, PFE1605w‐C interacted with recombinant band 3 with approximately 1.5 μM *K*_d_ (Fig. 6D). This result demonstrates the direct interaction of PFE1605w with a specific cytoskeletal protein, although we do not exclude the possibility that PFE1605w partakes in a larger multiprotein complex.

## Discussion

The remarkable number of exported PHIST proteins predicted and the dramatic lineage‐specific proliferation of this multigene family in *P. falciparum* only (Sargeant *et al*., 2006) suggest an important role for PHIST proteins in host cell modifications. These modifications lead to the dramatic morbidity and mortality observed with this parasite. This observation is reflected in the number of recent publications showing that PHIST proteins are involved in altering host cell rigidity (Mills *et al*., 2007; Maier *et al*., 2008), binding erythrocyte components (Silva *et al*., 2005; Mills *et al*., 2007; Pei *et al*., 2007a; Parish *et al*., 2013; Proellocks *et al*., 2014), reducing cytoadherence under flow (Maier *et al*., 2008; Proellocks *et al*., 2014), mediating cell–cell communication (Regev‐Rudzki *et al*., 2013), cytoskeletal association (Tarr *et al*., 2014) and elevated transcript levels of some *phist* genes in patients (Daily *et al*., 2005; Mok *et al*., 2007; Claessens *et al*., 2012). Although it has been assumed that most PHIST proteins contain one or more interaction epitopes (Sargeant *et al*., 2006), to the best of our knowledge, no detailed characterization of protein interactions directly linked to the functional role of a PHIST protein has been reported.

Here, we have functionally characterized PFE1605w, which has been shown to bind to the ATS domain of PfEMP1, comigrates with PfEMP1 in space and time and localizes to Maurer's clefts and knobs (Oberli *et al*., 2014), although an alternative localization has been suggested (Proellocks *et al*., 2014). In addition to the well‐known and frequently used conditional post‐translational regulation using an FKBP DD, we applied a ‘knock‐sideways’ or ‘anchor‐away’ system (Haruki *et al*., 2008; Busch *et al*., 2009; Robinson *et al*., 2010; Xu *et al*., 2010). With this method, we took advantage of the rapalog‐induced heterodimerization of the FKBP12 and FRB domains to tether PFE1605w at Maurer's clefts, the transient location for a variety of parasite proteins destined to the iRBC membrane and surface. The tethering technique is a powerful way of revealing the function of an exported protein in host cell refurbishment and helps to dissect the role of these proteins within the export pathway. Both methods, tethering of PFE1605w at Maurer's clefts and protein destabilization, confirmed that mislocalization or depletion of PFE1605w did not result in reduced surface exposed PfEMP1, suggesting no obvious role for PFE1605w in PfEMP1 transport. At the same time, however, both methods of PFE1605w depletion from knobs resulted in large reduction of cytoadherence to CD36.

The different levels of reduction in parasite cytoadherence to specific endothelial receptors upon PFE1605w depletion suggest a highly specialized role for this protein. Previously, we tested six PfEMP1 ATS variants for binding the PHIST domain of PFE1605w (Oberli *et al*., 2014) and revealed up to 25‐fold differences in binding affinities. This suggests that sequence variation in ATS has optimized PFE1605w for binding to a PfEMP1 subset and that perhaps other PHIST proteins might have coevolved with specific ATS domains to create interaction pairs with maximum binding strength. In this simplified model, differences in the PFE1605w–ATS binding affinity might be expected to account for differences in the cytoadherence phenotype upon PFE1605w depletion.

Our assays partly support this model of PFE1605w function, as evidenced by the lack of an effect of PFE1605w depletion on cytoadherence observed for CSA‐binding parasites, where the ATS‐C fragment of the dominantly expressed PfEMP1 variant (PFL0030c, VAR2CSA) had a very weak binding affinity to the PFE1605w PHIST domain (*K*_d_ ~ 90 μM). In contrast, the ATS‐C fragments of PfEMP1 variants most often found in ICAM‐1‐binding parasites, PF07_0050 and PFL0020w, have up to sixfold higher PFE1605w affinity, and ICAM‐1 parasite cytoadherence is reduced by 30% upon PFE1605w depletion.

The complete picture, however, is more nuanced. Co‐IP assays coupled with MS robustly detected the *in vivo* association of PFE1605w with two mini‐PfEMP1 constructs encompassing the ATS‐C fragment of two PfEMP1 variants. These variants represent the two main subtypes of PfEMP1 ATS, groups A (PF08_0141) and B (PFF0010w). The *in vitro* affinity of these ATS domains for the PFE1605w PHIST, however, varies by more than 10‐fold. We also observed that the ATS‐C fragment of the PfEMP1 variant dominantly expressed in CD36‐binding parasites, PFD0615c, displays essentially no direct affinity for PFE1605w, despite the large decrease in CD36 cytoadherence upon PFE1605w depletion. The result seems to contradict the simple PFE1605w functional model presented above.

**Figure 7:**
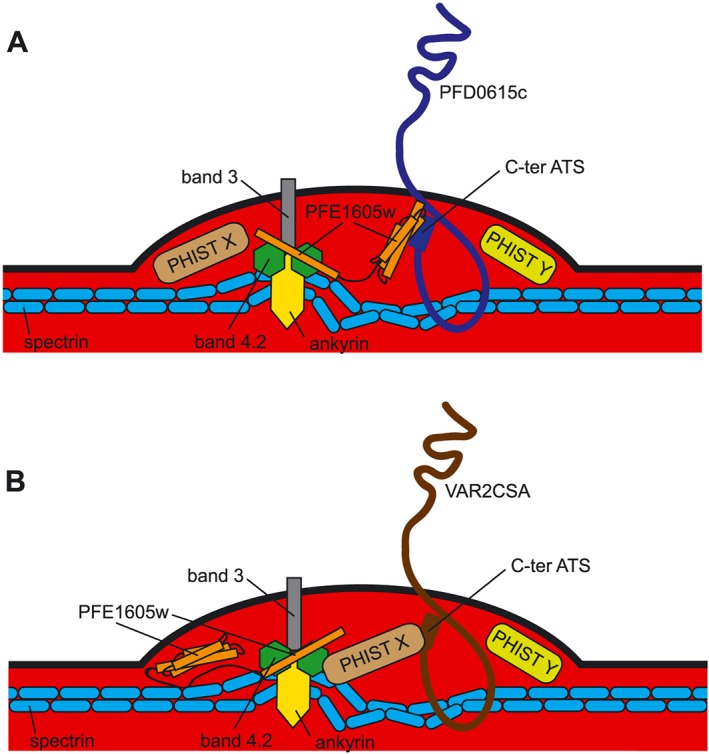
Schematic representation of the proposed functional role of PFE1605w within the iRBC knob structure. A. Both Co‐IP experiments and *in vivo* experiments showed that the PHIST domain of PFE1605w binds the C‐terminal part of different ATS domains and the C‐terminal part of PFE1605w targets the host band 3 and junctional complexes, thus making it an anchoring molecule between PfEMP1 and the host cytoskeleton. B. As various PHIST domains interacted with the same PfEMP1 epitope but with different affinities, it is conceivable that another PHIST protein might take over the function of PFE1605w depending on the surface exposed PfEMP1 molecule.

To reconcile these results, we must consider the following: PFE1605w directly binds the majority of ATS domains tested here and in previous studies (Oberli *et al*., 2014). This interaction is present *in vivo* for both strongly and weakly associated PFE1605w–ATS pairs. PFE1605w does not affect PfEMP1 transport and it colocalizes to knobs with PfEMP1 (Oberli *et al*., 2014). Indeed, the significant reduction in receptor binding upon tethering PFE1605w to Maurer's clefts strongly indicates that this protein exercises its functional role in knobs. There, PFE1605w is likely to be joined by (and might act together with) other ATS‐binding PHIST proteins such as PFI1780w (Mayer *et al*., 2012; Oberli *et al*., 2014), thereby accounting for the partial disruption of cytoadherence upon PFE1605w depletion. Further, in certain PfEMP1 variants, such as the CD36‐binding PFD0615c, the PFE1605w–ATS interaction might be mediated or strongly reinforced by other PHIST proteins. Because most of the PHIST proteins are expressed simultaneously, it seems that partnering must occur in the cytosol of the host or directly at the periphery (Fig. 7). Transcriptome analyses of all selected cell lines grown with or without shield excluded a possible upregulation of certain PHIST (data not shown).

To date, only a few direct interactions of exported proteins with cytoskeletal components of erythrocytes have been described and confirmed, e.g. KAHRP (Pei *et al*., 2005; Weng *et al*., 2014), PfEMP3 (Pei *et al*., 2007b), RESA (Pei *et al*., 2007a) and MESA (Magowan *et al*., 2000). Both KAHRP and PfEMP3 are required for correct trafficking and functional PfEMP1 display on the erythrocyte surface (Crabb *et al*., 1997; Waterkeyn *et al*., 2000), while PFE1605w is not. Co‐IP experiments with the full‐length PFE1605w–HA fusion protein identified a number of host integral membrane proteins and components of the erythrocyte cytoskeleton as putative binders, and fluorescence polarization experiments confirmed the direct interaction of the PFE1605w C‐terminus with the cytosolic domain of band 3. Thus, we have now shown the presence of two interaction epitopes within PFE1605w, at its PHIST domain and the C‐terminus, making it an anchoring molecule between PfEMP1 and the host cytoskeleton. The findings are consistent with previous assays suggesting an association of PFE1605w C‐terminal fragments with erythrocyte‐derived inside‐out vesicles (Proellocks *et al*., 2014). Both co‐IP and *in vitro* experiments suggest that PFE1605w, and thus PfEMP1, targets the host's band 3 and junctional complexes, while, interestingly, PFE1605w was the only parasite protein detected. The next step would be to map the exact interaction epitopes of PFE1605w with band 3 and possibly other cytoskeletal proteins. In addition to elucidating the complex that anchors PfEMP1 to the cytoskeleton, it would be valuable to study whether other PHIST proteins might bind PfEMP1 variants, in particular VAR2CSA, where CSA cytoadherence was not reduced upon depletion of PFE1605w. The PHIST interactome invites further studies to fully understand the remodelling of the host cell leading to pathology.

In summary, we show that the PHIST protein PFE1605w binds not only to PfEMP1 but also to members of band 3 and junctional complexes of the host cell. PFE1605w, however, plays no role in the transport of PfEMP1. We also show that various PfEMP1 molecules interact differently with PFE1605w and binding to endothelial receptors is partially disrupted upon conditional knock‐down or misplacement of PFE1605w. A profound analysis of other exported PHIST proteins and their interaction partners should help to reveal key components of the cytoadherence complex.

## Experimental procedures

### Parasite culture and transfection

*Plasmodium falciparum* 3D7 cell culture and transfection were performed according to standard procedures (Ljungström *et al*., 2008). Transfected parasites were grown in the presence of the indicated combinations of 10 nM WR99210 (Jacobs Pharmaceuticals, Cologne, Germany), 2.5 mg ml^−1^ blasticidin (Life Technologies, Zug, Switzerland), 625 nM Shield‐1 and 100 nM A/C Heterodimerizer (Clontech).

### Plasmid constructs

Primers 5′‐ATTTGGATCCATGAGGTTTACTAATTCATTATATTCG‐3′ and 5′‐ATATGCTAGCATTTTTTTTTTTATTTTCTTTTCCAGATTTG‐3′ were used to clone full‐length PFE1605w into pBcamR–3xHA (Flueck *et al*., 2009) via BamHI and NheI restriction sites. To fuse the FKBP DD to the C‐terminus of PFE1605w in 3D7 wild‐type parasites, a 785 bp flank of the 3′ end of PFE1605w was cloned into pARL‐DD via BglII and AvrII restriction sites by using the primers 5′‐ATATAGATCTTAACAGCAAATAGATTTT TATGGAG‐3′ and 5′‐ATATCCTAGGATTTTTTTTTTTATTTTCTTTTCCAGATTTG‐3′. MAHRP1 was cloned into mal7–mCherry–FRB (kindly provided by Tobias Spielmann (Grüring *et al*., 2011)) via XhoI and KpnI restriction sites by using the primers 5′‐ATATCTCGAGATGGCAGAGCAAGCAGC‐3′ and 5′‐CAGCGGTA CCATTATCTTTTTTTTCTTGTTCTAATTTTGC‐3′. Mini‐PfEMP1 constructs were synthesized (Fig. S4B) and cloned into pBcamR‐3xHA via NcoI and NheI restriction sites.

### Western blot analysis

Parasite proteins were obtained as previously described (Oberli *et al*., 2014), and samples were run on 12% w/v polyacrylamide bis‐Tris, 4–12% w/v polyacrylamide bis‐Tris or 3–8% w/v polyacrylamide Tris‐acetate NuPAGE gels (Invitrogen). Proteins were detected by using rabbit antibodies directed against the PFE1605w PHIST domain (α‐PFE1605w) (Pacific Immunology Inc.) (Fig. S1B), rabbit α‐HA (Roche 1:100), mouse α‐glyceraldehyde‐3‐phosphate dehydrogenase (α‐GAPDH) (1:20 000), rat α‐mCherry (Life Technologies; 1:1000) and mouse α‐ATS (1:500). PfEMP1 was extracted as described (Van Schravendijk *et al*., 1993) and detected with the mouse α‐ATS (1:500) antibody.

### Southern blot analysis

Genomic DNA of saponin‐lysed parasites was isolated as previously described (Beck, 2002). DNA was digested with AflII and XhoI restriction enzymes (New England Biolabs), separated on a 0.8% w/v agarose gel and transferred to a Amersham Hybond–N^+^ membrane (GE Healthcare). The blot was probed with [^32^P]‐dATP‐labelled h*dhfr* PCR fragments.

### Fluorescence microscopy

Immunofluorescence assays were performed on acetone‐fixed blood smears of infected parasite cultures (Spielmann *et al*., 2003) and blocked with 3% v/w BSA. Primary antibodies included rabbit α‐PFE1605w (1:200), mouse α‐KAHRP (1:200), mouse α‐RESA (1:250), rabbit α‐MESA (1:250), mouse α‐ATS (1:100), mouse α‐PfEMP3 (1:100), rabbit α‐MAHRP1 (1:200), rabbit α‐HSP70x (1:500) and rat α‐mCherry (Life Technologies; 1:200). Secondary antibodies (goat α‐rabbit Alexa 594, goat α‐mouse Alexa 594, goat α‐rabbit Alexa 488, goat α‐rat Alexa 594; Invitrogen) were incubated with 1 µg ml^−1^ 4,6‐diamidino‐2‐phenylindole (DAPI; Roche) at 1:200 dilution. Images were taken with a Zeiss LSM 700 confocal microscope (Carl Zeiss GmbH, Jena, Germany), with ×63 oil‐immersion lens (1.4 numerical aperture) and processed in photoshop cs6.

### Scanning electron microscopy

After knob selection and Percoll purification, the erythrocytes/iRBCs were fixed in 2% v/v glutaraldehyde in phosphate buffer for 1 h at room temperature. After three washes in PBS, the samples were transferred to coverslips preliminary coated with poly‐l‐lysine (Sigma), dehydrated in increasing concentration of ethanol (10% v/v, 25% v/v, 50% v/v, 75% v/v, 90% v/v and 2× 100% v/v, 10 min each) and dried at the critical point. Finally, coverslips were mounted onto stubs, sputtered with 5 nm platinum (LEICA EM ACE600) and imaged at 5 kV with a SEM Versa 3D (FEI). The micrographs were coloured in photoshop cs6.

### Trypsin cleavage assay

For trypsin cleavage, Percoll‐purified trophozoite stage parasites were incubated either in L‐(tosylamido‐2‐phenyl) ethyl chloromethyl ketone‐treated trypsin (Sigma, 100 µg ml^−1^ in PBS) or in trypsin and 1 mg ml^−1^ soybean trypsin inhibitor (Sigma, 1 mg ml^−1^ in PBS) for 15 min at 37°C. The digest was stopped by the addition of soybean trypsin inhibitor to a final concentration of 1 mg ml^−1^. PfEMP1 extraction and subsequent analysis was done as previously described (Van Schravendijk *et al*., 1993; Waterkeyn *et al*., 2000).

### Selection for receptor binding with recombinant protein

Subpopulations of parasites were selected by panning the parental parasite cell line (3D7) over purified human recombinant CD36 (50 µg ml^−1^), CD31 (50 µg ml^−1^), ICAM‐1 (50 µg ml^−1^), Thrombospondin‐1 (50 µg ml^−1^), endothelial protein C receptor (50 µg ml^−1^) and CSA (20 µg ml^−1^) according to Ockenhouse *et al*. (1991). Recombinant proteins were dissolved in double‐distilled H_2_O to the indicated final concentration and absorbed to a six‐well tissue culture plate (Falcon 353045, Corning, NY, USA) overnight at 4°C. The wells were blocked with 1% w/v BSA in RPMI medium for 1 h at 37°C. and the parasite culture was added for 2 h with a gentle shake of the tissue culture plate every 15 min. Unbound parasites were removed by five gentle washes with RPMI‐Hepes and uninfected RBCs (5% haematocrit) were added. After 24 h of incubation allowing late‐stage parasites to release merozoites to invade new RBCs, the newly invaded RBCs were transferred into continuous cell culture. The panning procedure was repeated four times prior to RNA isolation and cytoadhesion assays.

### Cytoadhesion assay

Purified recombinant protein was spotted on wells of an eight‐chamber polystyrene vessel tissue culture‐treated glass slide (Falcon, Big Flats, NY, USA) with concentrations as indicated and coated overnight at 4°C to allow proteins to absorb to the surface. The wells were blocked with 1% w/v BSA in RPMI medium for 1 h at 37°C. Selected parasite cell lines were split and cultured separately with or without 500 nM Shield‐1 for 96 h. Parasites were washed twice with RPMI‐Hepes and spotted onto immobilized recombinant protein and cultured for 2 h under continuous and simultaneous shaking (140 r.p.m., proBlot 25 Rocker; Labnet International Inc., NY, USA) (105 r.p.m., Lab‐Therm LT‐W, Kühner, Switzerland) at 37°C. Non‐bound erythrocytes were removed by gently flooding each well with RPMI‐Hepes six times with simultaneous shaking for 2 min. Bound iRBCs were fixed with 2% v/v glutaraldehyde in RPMI‐Hepes overnight and stained with Giemsa for 1 h and microscopically quantified. Results are shown as mean number of parasites bound per square millimetre and normalized to 1% parasitaemia.

### Quantitative PCR for *P. falciparum* erythrocyte membrane protein 1 expression

Synchronized cultures of PFE1605w–DD expressing parasites preselected to bind CD36, ICAM‐1 or CSA were split and cultured 96 h in the presence (+) or absence (−) of Shield‐1, and ring‐stage parasites were used for *var* transcript profiling, as previously described (Dahlbäck *et al*., 2007). Transcript abundance of each 3D7 *var* gene was determined relative to internal control transcripts by qPCR by using gene‐specific primers and complementary DNA synthesized from total RNA extracted from pelleted infected erythrocytes dissolved in TRIzol.

### Recombinant protein expression

Codon‐optimized genes encoding the ATS‐C fragments of PfEMP1 variants (Fig. S4A) were cloned in a modified pET‐16 vector (Merc Millipore). Gene fragments coding for amino acids 300–528 of PFE1605w (PFE1605w‐C) or amino acids 1–379 of human erythrocytic band 3 were cloned in a pFloat2 vector (Rogala *et al*., 2015), which provides an N‐terminal His_6_ tag.

Purification and fluorescent labelling of ATS‐C and PFE1605w‐C was performed as previously described (Mayer *et al*., 2012); briefly, clones were transformed in *Escherichia coli* strain BL21(DE3), grown in Luria–Bertani medium and protein expression was induced with 0.1 mM isopropyl β‐d‐1‐thiogalactopyranoside. Cells were lysed by sonication, and proteins were purified from lysate supernatants by using metal‐affinity, ion‐exchange and size‐exclusion chromatography. Fluorescent labelling was performed by *N*‐(5‐fluoresceinyl)maleimide (5‐FAM; Invitrogen) conjugating to a single cysteine residue at the protein N‐terminus that was added during cloning. Labelled ATS‐C and unreacted dye were separated by size‐exclusion chromatography. Protein identity and 5‐FAM labelling was confirmed by electrospray ionisation (ESI) MS.

Purification of the PFE1605w PHIST domain and the cytosolic band 3 domain was performed as previously described (Zhang *et al*., 2000; Oberli *et al*., 2014).

### Fluorescence polarization binding assays

Fluorescence polarization measurements were recorded at 20°C by using a CLARIOStar fluorimeter (BMG Labtech; *λ*_ex_ = 485 nm, *λ*_em_ = 520 nm). Five hundred nanomolar 5‐FAM‐labelled ATS‐C variants in 50 mM NaCl, 20 mM Na_2_HPO_4_ pH 6.5 buffer were titrated with defined concentrations of PFE1605w PHIST domain in the same buffer. For the band 3–PFE1605w‐C interaction, 0.5 μM 5‐FAM‐labelled PFE1605w‐C in 50 mM NaCl, 20 mM Na_2_HPO_4_ pH 7.0 buffer was titrated with unlabelled band 3. Changes in fluorescence polarization were fitted by using a single binding model in the program origin (OriginLab).

### Coimmunoprecipitation experiments

Three hundred millilitres of cell culture (5% haematocrit, 5‐8% parasitaemia) of 3D7 parasites or 3D7 parasites episomally expressing PFE1605w–3xHA/mini‐PfEMP1 was cross‐linked in 1% v/v formaldehyde. The reaction was stopped after 10 min by adding 2.5 M glycine. Immunoprecipitation was performed as previously described (Dietz *et al*., 2014). For the Co‐IP experiments with the mini‐PfEMP1 fusion protein, Pierce α‐HA magnetic beads (Thermo Scientific) were used. For the reverse Co‐IP with α‐band 4.2 antibodies, Dynabeads Protein G were used together with the cross‐linking reagent BS3 to avoid coelution of antibodies, according to the manufacturer's protocol (Life Technologies). The eluted fraction was analysed on a 4–12% w/v polyacrylamide bis‐Tris gel (Invitrogen) and fractions of it or TCA precipitated pellets were sent to the central core facility for LC‐MS/MS analysis.

## Conflict of interest

The authors declare no conflict of interest.

## Author contributions

AO, LZ and SR performed the cell biological experiments; FB performed the electron microscopy experiments; MEB, JLD and EEC performed the biophysical interaction experiments; TL analysed the *var* gene expression and AO, JV and HPB conceived the experiments and wrote the paper.

## Supporting information


**Fig. S1.** Plasmid maps, Southern blot and Western blots of extracts from cell lines used in this study.
**Fig. S2.** Localization of well‐characterized exported proteins upon PFE1605w reduction.
**Fig. S3.** Reduced levels of PFE1605w do not alter knob formation.
**Fig. S4.** ATS‐C and mini‐PfEMP1 constructs.

Supporting info item

Supporting info item

Supporting info item

Supporting info item
